# Social frailty independently predicts 9-year mortality among community-dwelling older adults in Mexico: validation of a brief social frailty index

**DOI:** 10.3389/fpubh.2026.1874301

**Published:** 2026-07-20

**Authors:** Liliana Giraldo-Rodríguez, Lorena Parra-Rodríguez, Teresa Álvarez-Cisneros, Marcela Agudelo-Botero

**Affiliations:** 1 Policy, Population, and Health Research Center, School of Medicine, National Autonomous University of Mexico, Mexico City, Mexico; 2 Research Department, Instituto Nacional de Geriatría, Ciudad de México, Mexico; 3 Internal Medicine Department, Hospital Médica Sur, Ciudad de México, Mexico

**Keywords:** all-cause mortality, Latin America, longitudinal study, older adults, psychometrics, social determinants of health, social frailty

## Abstract

**Introduction:**

Social frailty, defined as deficits in social resources, roles, and engagement, is an emerging risk dimension. However, evidence on its measurement and prognostic value in Latin America is limited.

**Methods:**

Using nationally representative data from the Mexican Health and Aging Study (2012-2021), we adapted a five-item Social Frailty Index (SFI) and evaluated its psychometric properties and association with 9-year all-cause mortality in 5,400 community-dwelling adults aged ≥60 years.

**Results:**

At baseline, 38.9% were pre–frail (SFI = 1) and 25.2% were frail (SFI ≥ 2). Mortality increased progressively across categories. In fully adjusted Cox models, pre-frailty (HR = 1.21, 95%CI: 1.06–1.37) and frailty (HR = 1.56, 95%CI: 1.36–1.79) independently predicted higher mortality risk.

**Discussion:**

The SFI, adapted to available MHAS data, demonstrated prognostic relevance. This provides initial evidence supporting further research on social frailty assessment in the region, including cultural adaptation and external validation.

## Introduction

Frailty is a well-established multidimensional geriatric syndrome characterized by increased vulnerability to adverse outcomes such as disability, hospitalization, and death (1, 2). Conceptual frameworks describe frailty as arising from deficits across interrelated domains—most commonly physical, psychological, and social—each contributing independently to adverse health trajectories (2, 3). Although physical frailty has traditionally received the greatest attention, the social domain is increasingly recognized as a critical and distinct component of the frailty construct (4).

Social frailty refers to a continuum of risk related to the loss, or potential loss, of social resources, roles, activities, and self-management capacities necessary to meet basic social needs over the life course (5). It manifests through diminished social participation, reduced contact, loss of perceived usefulness, or weakened social networks (6, 7). Importantly, social frailty is not merely a consequence of physical decline but may also precede physical manifestations and independently predict adverse outcomes such as disability and mortality (7–9).

Measurement approaches to social frailty have evolved from deficit accumulation models (4, 10, 11) toward multidimensional frameworks grounded in social functioning, social resources, and the fulfilment of basic social needs (4, 12). This theoretical shift has informed the development of brief screening instruments such as the Makizako Social Frailty Index (6, 8), the Social Frailty Index (13), the HALFT scale (5), and the 4-item social frailty screening questionnaire (7). Establishing sound psychometric properties for these instruments is essential, as systematic reviews reveal substantial variation in reported social frailty prevalence across populations and settings (14, 15).

Longitudinal evidence and meta-analyses consistently identify social frailty as an independent predictor of all-cause mortality, even after adjustment for health and sociodemographic factors (16, 17). These associations have been documented primarily in Asia, Europe, and North America (5, 7, 9, 18), whereas evidence from Latin America remains limited. As a result, the prevalence, structure, and prognostic relevance of social frailty in this region are not yet well characterized.

Mexico is undergoing rapid population ageing in the context of persistent socioeconomic and gender inequalities that shape health trajectories in later life. Although physical and cognitive frailty have been widely studied in this setting, social frailty has not been systematically operationalized in national surveys, constraining empirical evidence on its population distribution and health consequences. Addressing this gap is particularly relevant given the social and structural conditions influencing ageing in Mexico.

This study aimed to adapt and psychometrically evaluate a brief Social Frailty Index (SFI) using data from the Mexican Health and Aging Study, informed by Makizako's social frailty framework and Social Production Function (SPF) theory (12, 19–21). We estimated the prevalence of social frailty and examined its association with all-cause mortality over nine years of follow-up. We hypothesized that socially prefrail and frail older adults would exhibit a higher risk of mortality, independent of sociodemographic characteristics, comorbidities, and baseline health status.

## Materials and methods

### Study design and data source

This analysis draws on longitudinal data from the Mexican Health and Aging Study (MHAS) (22), a prospective, nationally representative panel study of community-dwelling older adults in Mexico, initiated in 2001 with follow-up waves conducted in 2003, 2012, 2015, 2018, and 2021. For the present study we used data from the last four waves (2012–2021) as there is a longer interval between 2003–2012, during which a refresher sample was incorporated in 2012 to maintain national representativeness.

The MHAS protocol was approved by the relevant institutional review boards, and all participants provided informed consent. The present secondary analysis used publicly available de-identified data and was exempt from additional ethical review.

### Study cohort

From the total of 15,723 individuals interviewed in the 2012 wave, 10,170 were aged 60 years or older and were therefore eligible for the present study. From this eligible population, we included participants with complete data on the Social Frailty Index and mortality follow-up, yielding an analytic sample of 5,400 participants. A total of 4,770 eligible participants were excluded because of missing information on one or more SFI items or on mortality follow-up required for the analyses.

### Outcome and mortality time

The outcome of interest was all-cause mortality. Mortality time was calculated as the number of years from the baseline interview (2012) to the date of death or censoring. For deceased participants, time-to-event was defined as the interval between the baseline interview and the month of death, as reported by next-of-kin. To avoid zero follow-up time for deaths occurring in the interview month, a midpoint imputation of 0.5 months was applied (23). For survivors, follow-up time was censored at the date of the 2021 interview, while for participants lost to follow-up, censoring occurred at the date of their last completed interview (2015–2018).

### Measurement of variables

#### Social frailty

Social frailty at baseline (2012) was assessed using a five-item Social Frailty Index (SFI) adapted from the instrument developed by Makizako and colleagues (8). The five domains and their operationalizations in the MHAS were:

- Social participation: Participants reporting engagement in religious, training, or social/sporting activities, or regular exercise (≥3 times/week) at least monthly.
- Social contact: Visits or communicates with friends or neighbors at least monthly.
- Social role: Feels helpful to friends/family through caring for children or adults, volunteering, performing maintenance/repairs or working for pay at least monthly.
- Living arrangement: Lives alone (1 = yes, 0 = no).
- Communication: Talks with someone every day (via in-person or phone/internet contact).

Each item was coded 1 if the social frailty-related deficit was present and 0 otherwise. Total scores (range 0–5) were categorized as: 0 = robust, 1 = prefrail, and ≥2 = frail.

#### Covariates

Cox models were adjusted for covariates in three domains: (1) Demographic: age (continuous), sex, and marital status (married/partnered vs. without partner); (2) Socioeconomic: years of schooling, household wealth (standardized z-score derived from housing characteristics and asset ownership), and place of residence (urban: ≥100,000 residents; rural: <100,000) (24); (3) Health conditions: cardiometabolic conditions (hypertension, diabetes, and heart attack or stroke) were grouped into mutually exclusive categories reflecting single and combined diagnoses (25, 26). Additional conditions included respiratory illness, arthritis, cancer, and recent hospitalization.

### Statistical analysis

We first conducted descriptive analyses of baseline demographic and health characteristics. Sampling weights were not applied, as the primary objective was etiologic inference and internal validity rather than population-level estimation. Continuous variables are reported as means and standard deviations (SD), and categorical variables as frequencies and percentages. Differences across Social Frailty Index (SFI) categories and between survivors and deceased participants were assessed using the Kruskal–Wallis and Mann–Whitney tests for continuous variables, and χ^2^ tests for categorical variables.

#### Psychometric validation

The internal consistency of the five dichotomous SFI items was evaluated using the Kuder–Richardson 20 (KR-20) coefficient and tetrachoric correlations. Dimensionality was examined through exploratory factor analysis of the tetrachoric correlation matrix. Because the SFI was treated as a brief composite index of heterogeneous social deficits, internal consistency and dimensionality results were interpreted to assess whether the items behaved as a unidimensional reflective scale or as distinct indicators contributing to a pragmatic risk index.

#### Predictive validation

We assessed the predictive validity of the SFI for all-cause mortality. Kaplan–Meier curves estimated unadjusted survival probabilities by SFI category; differences were tested with the log-rank statistic. Cox proportional-hazards models with robust standard errors examined the association between social frailty and all-cause mortality in four hierarchically adjusted models: Model 0 (unadjusted); Model 1 (+ demographic: age, sex, marital status); Model 2 (+ socioeconomic: education, wealth, rurality); Model 3 (+ health conditions). Hazard ratios (HR) and 95% confidence intervals (CI) were reported. Model discrimination was assessed using Harrell's C-index and Gönen and Heller's K statistic. The proportional hazards assumption was evaluated using Schoenfeld residuals, and time-varying effects were modeled through interactions with log-transformed time. All analyses were conducted using Stata 17 (StataCorp, College Station, TX, USA) (27).

## Results

Among the 10,170 eligible participants aged 60 years or older, 5,400 had complete data on the Social Frailty Index and mortality follow-up and were included in the analytic sample, while 4,770 were excluded because of missing information (see Supplementary Table S1). Compared with included participants, excluded participants were older (71.8 ± 8.7 vs. 69.1 ± 7.0 years), had fewer years of schooling (4.0 ± 4.3 vs. 5.4 ± 4.5 years), lower household wealth, a higher proportion of unmarried individuals (39.5% vs. 33.8%), and a higher proportion of rural residence (47.9% vs. 37.4%). The distribution of cardiometabolic multimorbidity also differed between groups. Respiratory illness, arthritis, and hospitalization in the previous year did not differ significantly between groups.

In the analytic sample, 1,939 participants (35.9%) were classified as robust according to the Social Frailty Index, 2,101 (38.9%) as prefrail, and 1,360 (25.2%) as frail at baseline. During the 9-year follow-up period, mortality increased progressively with the level of social frailty: 403 robust participants (20.8%), 574 prefrail participants (27.3%), and 557 frail participants (41.0%) died (*p* < 0.001).

The distribution of the individual items constituting the social frailty index is shown in Table 1. The most prevalent deficits were reduced social participation, reported by 36.5% of participants, and a lack of perceived usefulness to others (27.7%). Less common items were living alone (5.9%) and not communicating daily with family or friends (6.8%). Overall, reduced social participation and perceived usefulness were the most prevalent dimensions of social frailty in the cohort.

**Table 1:** Prevalence of individual social frailty index items and frailty status and internal consistency of the five Social frailty index items (*n* = 5,400).

Social frailty items	Total *n* = 5,400	Item–test correlation	Item–rest correlation	Average inter-item correlation	α if item deleted
No social participation	1,969 (36.5)	0.489	0.079	0.029	0.107
No social contact	1,018 (18.9)	0.462	0.047	0.040	0.143
No social role	1,494 (27.7)	0.537	0.138	0.010	0.038
Lives alone	321 (5.9)	0.426	0.007	0.054	0.186
No communication	368 (6.8)	0.469	0.056	0.037	0.133
**Test scale (KR-20)**				**0.034**	**0.150**
Social frailty index					
Robust	1,939 (35.9)				
Prefrail	2,101 (38.9)				
Frail	1,360 (25.2)				

Social frail individuals were significantly older, less educated, and economically disadvantaged, with lower total wealth. They were also more likely to be unmarried and to report poorer health status (*p* < 0.001) than robust participants. A clear gradient was observed in health outcomes, where the prevalence of cardiometabolic multimorbidity and the rate of recent hospitalizations increased progressively with the severity of social frailty. This cumulative disadvantage was ultimately reflected in mortality: while 28.4% of the cohort died during follow-up, mortality rates sharply increased from 20.8% in the robust group to 41.0% among frail individuals (*p* < 0.001) (Table 2).

**Table 2:** General characteristics at the 2012 baseline of the population aged 60 years and older by the Social Frailty Index.

Characteristics	Total *n* = 5,400 Mean ±SD or *n* (%)	Robust *n* = 1,939 Mean ±SD or *n* (%)	Prefrail *n* = 2,101 Mean ±SD or *n* (%)	Frail *n* = 1,360 Mean ±SD or *n* (%)	*p*-value
Deceased	1,534 (28.4)	403 (20.8)	574 (27.3)	557 (41.0)	<0.001
Age (years)	69.1 ± 7.0	67.4 ± 5.9	68.9 ± 6.7	71.6 ± 8.2	<0.001
Sex (men)	2,343 (43.4)	918 (47.3)	894 (42.6)	531 (39.0)	<0.001
Years of schooling (years)	5.4 ± 4.5	6.0 ± 4.7	5.3 ± 4.5	4.6 ± 4.2	<0.001
Unmarried	1,827 (33.8)	525 (27.1)	722 (34.4)	580 (42.7)	<0.001
Total household wealth (MXN)	1,012,313.4 ± 1,465,022	1,096,289.6 ± 1,560,295.2	1,003,626.5 ± 1,506,115.1	906,005.5 ± 1,236,229.1	<0.001
Rurality	2,018 (37.4)	768 (39.6)	768 (36.6)	482 (35.4)	0.032
Cardiometabolic multimorbidity					
None	2,116 (39.3)	825 (42.6)	838 (40.0)	453 (33.4)	<0.001
Only hypertension	1,666 (30.9)	603 (31.2)	649 (31.0)	414 (30.5)	
Only diabetes	440 (8.2)	158 (8.2)	180 (8.6)	102 (7.5)	
Only heart attack (HA) or stroke	59 (1.1)	21 (1.1)	23 (1.1)	15 (1.1)	
Hypertension + diabetes	816 (15.2)	243 (12.6)	303 (14.5)	270 (19.9)	
Hypertension + HA or stroke	154 (2.9)	45 (2.3)	56 (2.7)	53 (3.9)	
Diabetes + HA or stroke	26 (0.5)	13 (0.7)	4 (0.2)	9 (0.7)	
Hypertension + diabetes + HA or stroke	109 (2.0)	27 (1.4)	41 (2.0)	41 (3.0)	
Respiratory illness	358 (6.6)	121 (6.2)	145 (6.9)	92 (6.8)	0.678
Arthritis	915 (17.0)	293 (15.1)	355 (16.9)	267 (19.7)	0.003
Cancer	146 (2.7)	52 (2.7)	49 (2.3)	45 (3.3)	0.225
Hospitalization past year	685 (12.7)	193 (10.0)	268 (12.8)	224 (16.5)	<0.001

Missing data for: education (*n* = 27), hypertension (*n* = 8), diabetes (*n* = 5), heart attack (*n* = 5), stroke (*n* = 1), respiratory illness (*n* = 6), arthritis (*n* = 7), cancer (*n* = 7), and hospitalization past year (*n* = 5). A χ^2^ test was used for categorical variables and a Kruskal-Wallis test for continuous variables.

### Internal consistency and dimensionality of the Social Frailty Index

The Social Frailty Index (SFI) comprised five binary items assessing distinct social domains: social participation, social contact, perceived social role, living arrangement, and daily communication. The scale showed low internal consistency (KR-20 = 0.15; average inter-item correlation = 0.034), with corrected item–rest correlations ranging from 0.007 to 0.138. Removal of individual items did not meaningfully improve the reliability coefficient (α if item deleted: 0.04–0.19). These psychometric properties suggest that the items capture heterogeneous social domains rather than a single homogeneous latent dimension. Accordingly, all five items were retained in the final index (Table 1).

Pairwise tetrachoric correlations among items were small to moderate (r = −0.09 to 0.21), indicating heterogeneity across social domains. The mean absolute correlation was 0.05 (Supplementary Table S2).

Exploratory factor analysis (EFA) of the tetrachoric correlation matrix (principal-factor method, unrotated) yielded small eigenvalues (0.40, 0.12, and 0.03), all well below unity, with no evidence of a dominant latent factor (Supplementary Table S3). All items displayed high uniqueness values (>0.81), indicating limited shared variance and supporting the interpretation of the index as formative rather than reflective (Supplementary Table S4).

The combined evidence from the reliability and factor analyses—characterized by low internal consistency, weak to moderate inter-item correlations, the absence of a dominant factor in the EFA, and high uniqueness values—suggests that the five SFI items capture distinct and only weakly correlated social domains. These findings limit the interpretation of the SFI as a unidimensional reflective scale of social frailty. Accordingly, in this study the SFI should be interpreted as a brief pragmatic index of heterogeneous social deficits adapted to the available MHAS items, rather than as a psychometrically homogeneous scale reflecting a single latent construct. Descriptive statistics by vital status are presented in Supplementary Table S5. Clear differences in baseline characteristics were observed between survivors and decedents. Compared with survivors, participants who died were older and more likely to be male, unmarried, and socioeconomically disadvantaged, with fewer years of formal education and lower household wealth. They also exhibited a substantially higher burden of cardiometabolic multimorbidity at baseline, particularly combinations involving diabetes and cardiovascular disease. In addition, a history of cancer and hospitalization in the year prior to baseline were significantly more prevalent among decedents.

### Survival analysis

**Figure 1:**
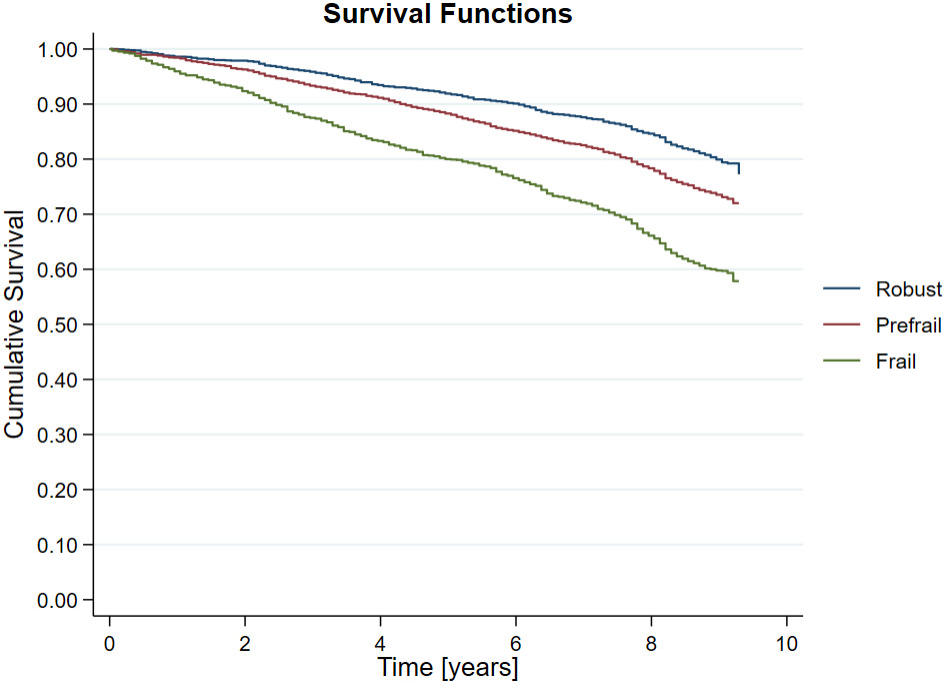
Kaplan–Meier survival curves showing cumulative survival according to baseline Social frailty index among community-dwelling older adults in Mexico (*n* = 5,400). Log-rank test *p* < 0.001.

Kaplan–Meier curves demonstrated a clear gradient in survival according to the Social Frailty Index (SFI) (Figure 1). Over the 9-year follow-up, cumulative survival was highest among robust participants, intermediate among prefrail individuals, and lowest among those classified as frail. The median survival time was not estimable for any group, as more than 50% of participants survived to the end of the follow-up period. However, the probability of survival declined progressively with increasing levels of social frailty. The log-rank test indicated statistically significant differences across the three curves (*p* < 0.001), consistent with a graded association between social frailty and mortality risk.

As shown in Table 3, cumulative survival decreased progressively with higher levels of social frailty. At 2 years, cumulative survival exceeded 90% in all groups; however, by 8 years it had declined to 84.6% among robust participants, 78.3% among prefrail participants, and 66.1% among frail participants. This graded reduction in survival is consistent with the widening separation of the Kaplan–Meier curves illustrated in Figure 1.

**Table 3:** Kaplan–Meier estimates of survival probability at two, four, six, and 8 years according to baseline Social Frailty Index (SFI) among community-dwelling older adults in Mexico (*n* = 5,400).

SFI	Participants at baseline *n* (100%)	At 2 years *n* (%)	At 4 years *n* (%)	At 6 years *n* (%)	At 8 years *n* (%)
**Robust**	1,939	1,899 (97.9)	1,815 (93.5)	1,749 (90.1)	1,644 (84.6)
**Prefrail**	2,101	2,025 (96.3)	1,919 (91.2)	1,792 (85.2)	1,653 (78.3)
**Frail**	1,360	1,264 (92.4)	1,135 (83.3)	1,047 (76.5)	906 (66.1)

Values correspond to the number and percentage of participants remaining under observation (i.e., not deceased or censored) at each follow-up year. Percentages represent the Kaplan–Meier survivor function, reflecting the estimated probability of survival at each time point.

### Social frailty and mortality: Cox regression results

In Cox proportional-hazards models, the Social Frailty Index (SFI) demonstrated a strong, graded association with 9-year all-cause mortality that persisted after sequential adjustment for demographic, socioeconomic, and health-related covariates (Table 4). In the unadjusted model, prefrail and frail individuals exhibited a significantly higher risk of mortality compared with robust participants (HR = 1.61, 95% CI: 1.23–2.13 and HR = 3.12, 95% CI: 2.39–4.06, respectively; *p* < 0.001). Although these associations were attenuated after full adjustment, they remained statistically significant, with prefrail and frail states associated with higher mortality risk (HR = 1.21, 95% CI: 1.06–1.37 and HR = 1.56, 95% CI: 1.36–1.79, respectively).

**Table 4:** Cox proportional hazards regression for unadjusted and adjusted models.

Characteristic	Model			
	Unadjusted Model (*n* = 5,400) HR (95% CI)	Model 1 Demographic (*n* = 5,400) HR (95% CI)	Model 2 Sociodemographic (*n* = 5,373) HR (95% CI)	Model 3 Sociodemographic + Health (*n* = 5,344) HR (95% CI)
Social frailty index				
Robust (ref.)	—	—	—	—
Prefrail	1.61 (1.23–2.13) *p = 0.001*	1.24 (1.10–1.41) *p = 0.001*	1.23 (1.09–1.41) *p = 0.001*	1.21 (1.06–1.37)*p = 0.005*
Frail	3.12 (2.39–4.06) *p = <0.001*	1.71 (1.49–1.95) *p = <0.001*	1.68 (1.47–1.93) *p = <0.001*	1.56 (1.36–1.79) *p = <0.001*
Demographic covariates				
Age (years)	—	1.08 (1.07–1.09) *p = <0.001*	1.08 (1.07–1.08) *p = <0.001*	1.08 (1.07–1.09) *p = <0.001*
Sex (Men)	—	1.30 (1.17–1.45) *p = <0.001*	1.34 (1.20–1.50) *p = <0.001*	1.40 (1.25–1.56) *p = <0.001*
Married	—	0.96 (0.86–1.08) *p = 0.528*	0.96 (0.86–1.08) *p = 0.530*	0.95 (0.84–1.06) *p = 0.343*
Socioeconomic covariates				
Years of schooling	—	—	0.99 (0.98–1.00) *p = 0.162*	0.99 (0.98–1.01) *p = 0.286*
Standardized total wealth	—	—	0.93 (0.88–0.99) *p = 0.021*	0.94 (0.88–0.99) *p = 0.029*
Rurality	—	—	0.98 (0.87–1.09) *p = 0.662*	1.06 (0.95–1.18) *p = 0.311*
**Cardiometabolic multimorbidity (ref**. = **none)**	
Only hypertension	—	—	—	1.02 (0.89–1.17) *p = 0.766*
Only diabetes	—	—	—	1.80 (1.50–2.15) *p = <0.001*
Only heart attack (HA) or stroke	—	—	—	1.36 (0.88–2.09) *p = 0.161*
Hypertension + diabetes	—	—	—	2.01 (1.73–2.32) *p = <0.001*
Hypertension + HA or stroke	—	—	—	1.35 (1.03–1.78) *p = 0.033*
Diabetes + HA or stroke	—	—	—	2.45 (1.46–4.11) *p = 0.001*
Hypertension + diabetes + HA or stroke	—	—	—	2.75 (2.10–3.60) *p = <0.001*
**Other health conditions**				
Respiratory illness	—	—	—	1.69 (1.27–2.24) *p = <0.001*
Cancer	—	—	—	2.06 (1.63–2.61) *p = <0.001*
Hospitalization past year	—	—	—	1.92 (1.54–2.39) *p = <0.001*
Time-varying coefficients				
Prefrail (SFI)	0.90 (0.77–1.06) *p = 0.208*	—	—	—
Frail (SFI)	0.81 (0.69–0.95) *p = 0.009*	—	—	—
Respiratory illness	—	—	—	0.78 (0.66–0.94) *p = 0.007*
Hospitalization past year	—	—	—	0.80 (0.70–0.92) *p = 0.002*

HR, hazard ratio; CI, confidence interval; HA, heart attack. Model 1: adjusted for age, sex, and marital status. Model 2: further adjusted for years of education, standardized household wealth, and rurality. Model 3: further adjusted for cardiometabolic multimorbidity, respiratory illness, cancer, and hospitalization in the prior year.

The inclusion of the SFI in the fully adjusted model resulted in a modest improvement in model discrimination, with Gönen and Heller's K increasing from 0.678 to 0.682 (ΔK = 0.004). Harrell's C showed a similarly small increase (0.714 to 0.718). Other strong predictors in the fully adjusted model included cardiometabolic multimorbidity (particularly combinations involving diabetes), cancer, and recent hospitalization.

Violations of the proportional hazards assumption were detected for respiratory illness, recent hospitalization, and frail status. Time-varying coefficient analyses suggested that the effects of respiratory illness and hospitalization, as well as the association between frail status and mortality, varied over follow-up time and were stronger earlier in follow-up. Nevertheless, the overall graded association between SFI categories and mortality remained evident in the adjusted Cox models.

## Discussion

This study provides an initial psychometric and prognostic evaluation of a brief Social Frailty Index for community-dwelling older adults in Mexico, using nationally representative longitudinal data from the MHAS. The findings demonstrate that the accumulation of deficits across distinct social domains is independently associated with increased long-term mortality. Consistent with its interpretation as a brief composite index of heterogeneous social deficits, the index exhibited low internal consistency while capturing non-redundant dimensions of social functioning, including participation, contact, perceived social role, living arrangement, and daily communication. These findings provide initial evidence on the psychometric behavior and prognostic relevance of a Makizako-informed social frailty index adapted to available MHAS items, addressing an important gap in the Latin American literature.

The observed associations are consistent with longitudinal evidence showing that social frailty predicts mortality across diverse sociocultural contexts. Meta-analyses and cohort studies from Asia, Europe, and North America have reported comparable graded relationships between social frailty and survival, even after adjustment for sociodemographic characteristics and baseline health (5, 7, 9, 16, 17, 28–30). The consistency of these findings across distinct social inequalities and institutional arrangements extends the generalizability of existing evidence to a Latin American context.

Within Mexico, these findings complement prior research based on the Mexican Health and Aging Study, which shows that socioeconomic disadvantage, multimorbidity, and functional limitations contribute to mortality risk in later life (31). The Social Frailty Index provided prognostic information beyond these established determinants, indicating that social frailty captures a distinct dimension of vulnerability not fully explained by clinical or demographic factors. By incorporating domains such as social participation and perceived usefulness, the index reflects aspects of everyday social functioning that extend beyond traditional measures of social isolation or living arrangements.

Potential pathways linking social frailty to mortality are supported by psychosocial and behavioral evidence. Reduced social participation and constricted relational networks may limit access to practical support for managing chronic conditions and undermine key psychosocial resources, such as a sense of belonging and perceived support (32). Furthermore, a diminished perceived social role directly compromises a sense of purpose and meaning (mattering), which is vital for mental and physical resilience. Limited daily contact may also delay recognition of health deterioration or restrict timely assistance during acute events. This erosion of psychosocial resources has tangible biological consequences (9, 29). Although mechanisms were not directly examined in this study, the persistence of the associations after extensive adjustment supports the interpretation of social frailty as an independent and cumulative risk dimension.

From a public health perspective, these results underscore the importance of incorporating social frailty into geriatric assessment and population ageing strategies in Mexico and similar settings. The Social Frailty Index offers a brief, theoretically grounded tool that may inform interventions aimed at strengthening social participation, promoting meaningful social roles, and improving daily social contact among older adults. Given the multidimensional nature of social frailty, effective responses are likely to require coordinated efforts across health services, social programs, and community-based organizations.

Strengths of this study include the use of a large, nationally representative cohort with long-term follow-up and the integration of psychometric assessment and prognostic evaluation within an explicit theoretical framework. Limitations include reliance on baseline social conditions, which precludes assessment of changes in social frailty over time. In addition, the analytic sample was restricted to participants with complete information on the Social Frailty Index and mortality follow-up. Eligible participants excluded because of missing information differed from those included in several baseline characteristics, including age, education, marital status, household wealth, rurality, and cardiometabolic multimorbidity. Therefore, potential selection bias due to missing data cannot be ruled out, and prevalence estimates and mortality associations should be interpreted with this limitation in mind. Additionally, some conceptual overlap between SFI items and sociodemographic covariates cannot be fully ruled out, which should be considered when interpreting the adjusted associations. Residual confounding also cannot be entirely excluded despite adjustment for sociodemographic and health-related factors. In particular, the use of secondary MHAS data limited the availability of broader psychosocial and functional covariates that may further confound the association between social frailty and mortality. Furthermore, the adaptation of the Makizako index to available MHAS items was not subjected to a formal expert review or consensus process to identify the most relevant indicators for the Latin American population. Relatedly, the ground-up development of a social frailty instrument specifically designed for Latin American populations whose social norms and behaviors surrounding ageing may differ from those of the Japanese context in which the original index was developed, remains a possibility that this study did not address. Although this constitutes a limitation, it may also be considered a strength of the present study, as the findings make evident the need for cultural adaptation and external validation of social frailty measures in the region. In particular, future studies should incorporate formal expert review to reach consensus on the most relevant social frailty indicators for Latin American populations. Future studies should also examine the index in relation to broader social, demographic, psychological, and functional variables to further assess its construct validity and applicability in Latin American populations.

## Conclusion

Social frailty represents a measurable and clinically relevant dimension of vulnerability among older adults in Mexico. This Makizako-informed Social Frailty Index, adapted to available MHAS items, showed prognostic relevance for long-term mortality. These findings provide initial evidence to support further research on social frailty assessment in Latin America, including expert review, cultural adaptation, and external validation in independent cohorts.

## Data Availability

Publicly available datasets were analyzed in this study. This data can be found here: https://www.mhasweb.org/Home/index.aspx.
