# Supplementary material for: Social frailty independently predicts 9-year mortality among community-dwelling older adults in Mexico: validation of a brief social frailty index
Source: Front Public Health. 2026 Jul 20;14:1874301. doi: 10.3389/fpubh.2026.1874301 (PMC13429442; doi:10.3389/fpubh.2026.1874301)
Supplement: Supplementary file 1 [file Supplementary_file_1.docx]

**Table S1.** Baseline characteristics of eligible participants included in and excluded from the analytic sample.

| **Characteristics** | **Excluded**  **n = 4,770**  **Mean ± SD**  **or n (%)** | **Included**  **n = 5,400**  **Mean ± SD**  **or n (%)** | **p-value** |
| --- | --- | --- | --- |
| Age (years) | 71.8 ± 8.7 | 69.1 ± 7.0 | <0.001 |
| Sex (men) | 2,310 (48.4) | 2,343 (43.4) | <0.001 |
| Unmarried | 1,884 (39.5) | 1,827 (33.8) | <0.001 |
| Years of schooling (years) | 4.0 ± 4.3 | 5.4 ± 4.5 | <0.001 |
| Total household wealth (MXN) | 766,314  ± 1,209,435.1 | 1,012,313.4  ± 1,465,022 | <0.001 |
| Rurality | 2,283 (47.9) | 2,018 (37.4) | <0.001 |
| Cardiometabolic multimorbidity |  |  | <0.001 |
| None | 2,118 (44.7) | 2,116 (39.3) |  |
| Only hypertension | 1,280 (27.0) | 1,666 (30.9) |  |
| Only diabetes | 365 (7.7) | 440 (8.2) |  |
| Only heart attack (HA) or stroke | 71 (1.5) | 59 (1.1) |  |
| Hypertension + Diabetes | 630 (13.3) | 816 (15.2) |  |
| Hypertension + HA or Stroke | 152 (3.2) | 154 (2.9) |  |
| Diabetes + HA or Stroke | 18 (0.4) | 26 (0.5) |  |
| Hypertension + Diabetes + HA or Stroke | 108 (2.3) | 109 (2.0) |  |
| Respiratory illness | 286 (6.0) | 358 (6.6) | 0.197 |
| Arthritis | 755 (15.9) | 915 (17.0) | 0.138 |
| Cancer | 99 (2.1) | 146 (2.7) | 0.040 |
| Hospitalization past year | 638 (13.4) | 685 (12.7) | 0.297 |

Missing data for: education (n=53), hypertension (n=23), diabetes (n=17), heart attack (n=14), stroke (n=10), respiratory illness (n=18), arthritis (n=20), cancer (n=17), and hospitalization past year (n=12). A χ^2^ test was used for categorical variables and a Kruskal-Wallis test for continuous variables. Excluded participants were restricted to individuals aged 60 years and older who were otherwise eligible for the study cohort but had “missing information on one or more SFI items or on mortality follow-up.

**Table S2.** Tetrachoric correlation matrix for the five Social Frailty Index items (n = 5 400).

|  | **No social participation** | **No social contact** | **No social role** | **Lives alone** | **No communication** |
| --- | --- | --- | --- | --- | --- |
| **No social participation** | 1.000 |  |  |  |  |
| **No social contact** | 0.052 | 1.000 |  |  |  |
| **No social role** | 0.210 | 0.110 | 1.000 |  |  |
| **Lives alone** | 0.021 | -0.090 | 0.078 | 1.000 |  |
| **No communication** | 0.007 | 0.110 | 0.159 | 0.025 | 1.000 |

**Table S3.** Eigenvalues from exploratory factor analysis of the Social Frailty Index items (n = 5,400).

| **Factor** | **Eigenvalue** | **Difference** |
| --- | --- | --- |
| **Factor 1** | 0.399 | 0.282 |
| **Factor 2** | 0.117 | 0.084 |
| **Factor 3** | 0.033 | 0.167 |
| **Factor 4** | -0.134 | 0.070 |
| **Factor 5** | -0.204 |  |

**Table S4.** Exploratory factor analysis (principal factors, unrotated) of the Social Frailty Index items

| **Variable** | **Factor 1** | **Factor 2** | **Factor 3** | **Uniqueness** |
| --- | --- | --- | --- | --- |
| **No social participation** | 0.296 | 0.094 | -0.113 | 0.891 |
| **No social contact** | 0.234 | -0.209 | -0.010 | 0.902 |
| **No social role** | 0.426 | 0.064 | -0.002 | 0.815 |
| **Lives alone** | 0.065 | 0.234 | 0.078 | 0.935 |
| **No communication** | 0.266 | -0.079 | 0.119 | 0.909 |

Note: Principal factor method, unrotated solution. Uniqueness represents unexplained variance for each item.

**Table S5.** Baseline characteristics of participants aged 60 years and older by vital status at the end of follow-up.

| **Characteristics** | **Total**  **n = 5,400**  **Mean ± SD**  **or n (%)** | **Alive**  **n = 3,866**  **Mean ± SD**  **or n (%)** | **Deceased**  **n = 1,534**  **Mean ± SD**  **or n (%)** | **p-value** |
| --- | --- | --- | --- | --- |
| Social Frailty Index |  |  |  | <0.001 |
| Robust | 1,939 (35.9) | 1,536 (39.7) | 403 (26.3) |  |
| Prefrail | 2,101 (38.9) | 1,527 (39.5) | 574 (37.4) |  |
| Frail | 1,360 (25.2) | 803 (20.8) | 557 (36.3) |  |
| Age (years) | 69.1 ± 7.0 | 67.6 ± 6.0 | 72.7 ± 8.1 | <0.001 |
| Sex (men) | 2,343 (43.4) | 1,597 (41.3) | 746 (48.6) | <0.001 |
| Unmarried | 1,827 (33.8) | 1,218 (31.5) | 609 (39.7) | <0.001 |
| Years of schooling (years) | 5.4 ± 4.5 | 5.6 ± 4.6 | 4.7 ± 4.3 | <0.001 |
| Total household wealth (MXN) | 1,012,313.4  ± 1,465,022 | 1,061,358.2  ± 1,561,353.2 | 888,710.11  ± 1,179,541.8 | <0.001 |
| Rurality | 2,018 (37.4) | 1,431 (37.0) | 587 (38.3) | 0.391 |
| Cardiometabolic multimorbidity |  |  |  | <0.001 |
| None | 2,116 (39.3) | 1,634 (42.4) | 482 (31.5) |  |
| Only hypertension | 1,666 (30.9) | 1,254 (32.5) | 412 (26.9) |  |
| Only diabetes | 440 (8.2) | 281 (7.3) | 159 (10.4) |  |
| Only heart attack (HA) or stroke | 59 (1.1) | 37 (1.0) | 22 (1.4) |  |
| Hypertension + Diabetes | 816 (15.2) | 493 (12.8) | 323 (21.1) |  |
| Hypertension + HA or Stroke | 154 (2.9) | 97 (2.5) | 57 (3.7) |  |
| Diabetes + HA or Stroke | 26 (0.5) | 11 (0.3) | 15 (1.0) |  |
| Hypertension + Diabetes + HA or Stroke | 109 (2.0) | 47 (1.2) | 62 (4.1) |  |
| Respiratory illness | 358 (6.6) | 231 (6.1) | 127 (8.3) | 0.002 |
| Arthritis | 915 (17.0) | 650 (16.8) | 265 (17.3) | 0.693 |
| Cancer | 146 (2.7) | 71 (1.8) | 75 (4.9) | <0.001 |
| Hospitalization past year | 685 (12.7) | 401 (10.4) | 284 (18.6) | <0.001 |

Missing data for: education (n=27), hypertension (n=8), diabetes (n=5), heart attack (n=5), stroke (n=1), respiratory illness (n=6), arthritis (n=7), cancer (n=7), and hospitalization past year (n=5). A χ^2^ test was used for categorical variables and a Kruskal-Wallis test for continuous variables.
